# Mechanical stress in Arabidopsis leaves orients microtubules in a 'continuous’ supracellular pattern

**DOI:** 10.1186/1471-2229-13-163

**Published:** 2013-10-18

**Authors:** Eveline Jacques, Jean-Pierre Verbelen, Kris Vissenberg

**Affiliations:** 1 Department Biology, Plant Growth and Development, University of Antwerp, Groenenborgerlaan 171, 2020 Antwerpen, Belgium

**Keywords:** Arabidopsis thaliana, Cell development, Leaf, Mechanical stress, Microtubules

## Abstract

**Background:**

Cortical microtubules form a dynamic network and continuously undergo shrinking (catastrophe), pausing and rebuilding (rescue). The advantage of such a dynamic system is that it may mediate appropriate responses in a short time span. Microtubules are known to play a pivotal role in determining the orientation of the cellulose microfibril deposition in the plant cell wall. The latter is a solid exoskeleton surrounding the protoplast. It forms the physical framework that interconnects most cells and has to bear the tensile stresses within the tissue. Here we describe the effect of externally applied pressure on microtubule organization in growing Arabidopsis leaves.

**Results:**

Confocal microscopy examination of transgenic plants bearing GFP-tagged TUA6 proteins led to the observation that application of an additional mechanical pressure on growing Arabidopsis leaves triggers an excessive bundling of microtubules within the individual cell. Besides, the microtubules seem to align in neighboring cells, creating a 'continuous’ supracellular pattern. This effect occurs within 3 hours after applied external force and is age-dependent, whereby only cells of leaves up to 19 days after sowing (DAS) are susceptible to the applied pressure.

**Conclusions:**

Upon externally applied pressure on developing Arabidopsis leaves, microtubules bundle and rearrange to form seemingly continuous supracellular patterns. As microtubules guide the cellulose synthase complexes, this observed reorganisation pattern probably affects the cellulose deposition, contributing to the reinforcement of the cell wall in a particular position to cope with the extra-applied pressure. The age-effect is reasonable, since younger cells, which are actively shaping their cell walls, are more vulnerable to altered mechanical stresses while in leaves older than 19 DAS, the walls are more robust and therefore can sustain the applied forces.

## Background

Compelling evidence supports the idea that biophysical aspects of the developing tissue are co-determinants of growth [1,2]. In plants, the cell walls form an interconnecting framework that glues all cells together and that has to bear all encountered stresses [3]. Tensions within the tissue are generated between the different cell layers (tissue stresses) [4-6] and between neighboring cells as the result of differential growth rates [2]. Furthermore, plant cell walls behave like an elastic vessel under pressure. Osmotic-driven water uptake in the vacuole of the protoplast generates a so called 'turgor pressure’ [5,7] that pushes against the wall.

To endure all these stresses, crystalline arrays of cellulose microfibrils are deposited into the wall. These microfibrils provide tensile strength, which depends on the orientation pattern and the overall matrix composition including other wall components like hemicelluloses and pectins [8]. Importantly, this fortified wall needs to acquire some plasticity during growth to deform and allow expansion. These events are tightly managed on cellular level by the directed guidance of the cellulose synthase (CESA) complexes, determining cellulose microfibril orientation, by modification of the hemicellulosic tethers between adjacent cellulose microfibrils [9], by targeted delivery/activation of extracellular proteins that modulate the wall properties and by alterations in the apoplastic pH [10].

Most of the abovementioned processes involve the cytoskeleton, a dynamic network that spans the cortical region and consists of microtubules and actin filaments. The latter deals with the targeted transport of exocytotic vesicles [11-13], while microtubules influence the positioning of newly deposited cellulose microfibrils by guiding the CESA complexes in the membrane [14-18].

Here, we demonstrate the response of microtubules upon applied pressure in pavement cells of the Arabidopsis leaf. The stress response involves a reorganization of the microtubule pattern within 3 hours of applied external force. This is characterized by a pronounced parallel alignment of the microtubules. Moreover, microtubules in adjacent cells seem to form an overarching pattern, creating the impression of a continuous and supracellular pattern. This response occurs in an age-dependent manner whereby only the younger leaves up to 19 days after sowing (DAS) are susceptible to the stress.

## Results

### Supracellular patterns

**Figure 1:**
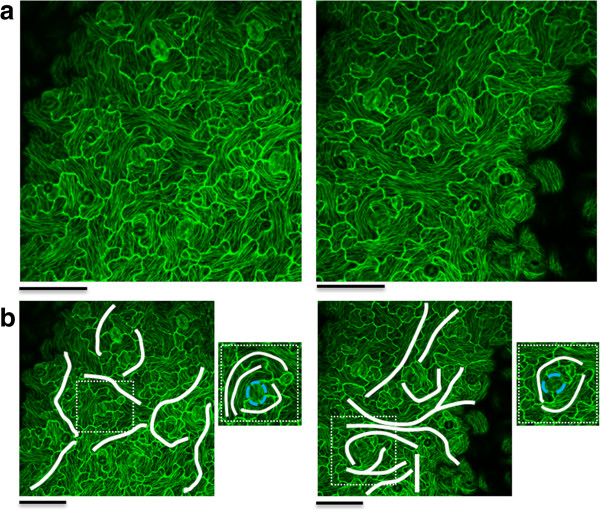
**Supracellular patterns.** Two example images of a supracellular microtubule stress pattern **(a)**. The continuous patterns visible on these images are marked in white lines on the images in **b**. The zoomed sections show circular supracellular patterns around stomata (marked by blue dotted line). (scale bar = 50 μm).

In order to apply an external stress on the leaf epidermis, we placed leaves from Arabidopsis plants expressing the microtubule marker TUA6-GFP between a microscopic slide and a cover slip and observed that prolonged incubation induces a strong alignment of microtubules within adaxial epidermal pavement cells. Figure 1a shows representative Z-stack projections after 3 hours of incubation. Intriguingly, this pattern seems to continue in neighbouring cells, implying a strong coordination on tissue level (Figure 1b). The patterns appear strongly around stomata as exemplified for the zoomed section images in Figure 1b. It needs to be mentioned that this transgenic line lacks the presence of trichomes.

### Microtubule stress response over time

**Figure 2:**
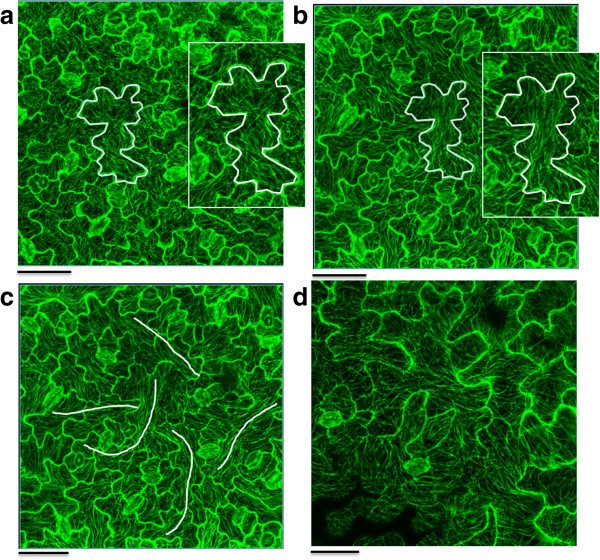
**Time series of microtubule patterns.** Image **a** shows the microtubule patterns in a leaf of 15 DAS at time point 0. Image **b** is taken at the same position but with a time interval of 3 hours and shows how microtubules were rearranged in parallel bundles within the cells. The zoomed sections show the altered microtubule patterns in more detail in the same cell at the initial time point **(a)** and after 3 hours **(b)**. The supracellular patterns that appear after 3 hours are marked with a white line on image **c**. (scale bar = 50 μm). Image **d** presents the microtubule patters after 3 hours for a leaf of 22 DAS.

To assess the time course of the microtubule response, microtubules were monitored over a period of 3 hours and microtubule patterns were recorded every 60 minutes. This was performed for leaves of different ages, ranging from 14 to 22 days after sowing (DAS). In all samples microtubules are organized in a rather random pattern at the initial time point (Figure 2a). After 180 minutes, however, a pronounced alignment of microtubules is visible as shown for pavement cells of 15 DAS in Figure 2b. Moreover, supracellular patterns can be observed as indicated by white lines on Figure 2c. At 22 DAS, however, no similar effect was visible, implying an age-dependent phenomenon (Figure 2d).

**Figure 3:**
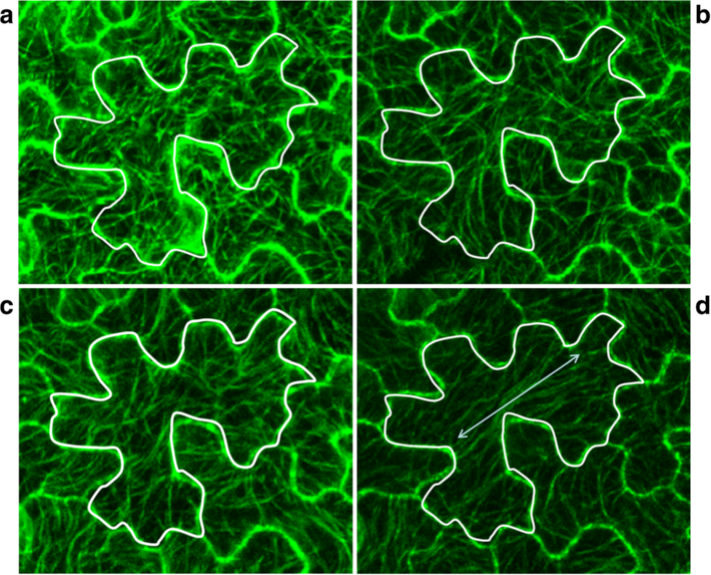
**Time series of the microtubule response.** Images of the microtubule patterns in a 15 DAS old leaf, after 0 **(a)**, 60 **(b)**, 120 **(c)** and 180 minutes **(d)**.

Figure 3 illustrates in more detail the changes in microtubule patterns over the 180 minute time period for a 15 DAS old leaf. This shows a dramatic change after 120 minutes, whereafter the microtubules align according to one axis in the cell (Figure 3d).

### Trigger of the stress response

**Figure 4:**
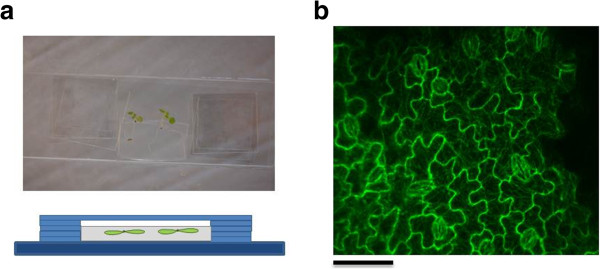
**Chambers with spacers in between microscopic slide and coverslip.** The set-up is illustrated in image **a**. The root of the plant is pushed in the medium while the leaves are freely present in the upper space, which is filled with distilled water. The lower image is an illustration of the side view, showing that the leaves are not pressured between the microscopic slide and the coverslip. The microtubule response after 6.5 hours is shown in **b** for a leaf of 19 DAS.

**Figure 5:**
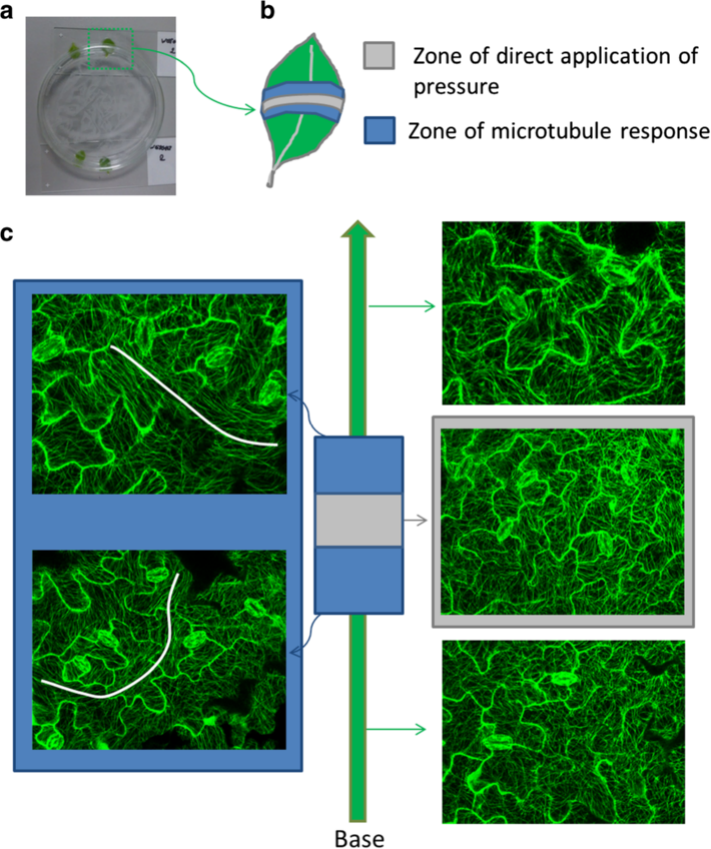
**Application of pressure at a specific location on the leaf.** External pressure is exerted by a up-side-down placed glass petri dish (50 mm) **(a)**. An 19 DAS old leaf can be divided in three distinct zones as shown on image **b**: zone of direct application of the pressure (grey), zone of microtubule response (blue) and the non-responding parts of the leaf (green). Representative images are shown in **c** for all three locations. (scale bar = 50 μm).

To identify the trigger that causes the realignment, different experimental conditions were tested (Table 1). As for normal confocal visualization the leaf is cut from the seedling and placed in water underneath a coverslip, the effect of the different individual manipulations was evaluated. The results show that no supracellular patterns could be observed in a leaf that is cut from the plant and that was left on a small water drop on a microscope slide for a period of 3 hours. Similarly, submerging the leaves in water did not induce stress patterns. Strong bundling of microtubules did only appear when the leaf was placed between the microscope slide and coverslip during 3 hours, even when it was still attached to the plant. Leaves of 20 DAS are variable in the response, with 2 out of 3 plants showing no remarkable changes in microtubule pattern (Table 1). This reflects the gradual age–dependent resistance to the applied stresses as described previously. To verify the anoxia effects caused by covering with the cover slip [19], special chambers were used with spacers in between the glass slide and the cover slip (Figure 4a). Imaging up to 6.5 hours did not reveal the previously described microtubule stress response (Figure 4b). These results provide strong evidence for the hypothesis that the pressure exerted by the coverslip is the initiator for the microtubule bundling and rearrangement towards 'continuous’ supracellular patterns. Indeed, directed application of external pressure induced the formation of strongly aligned microtubules that span the whole cell in leaves. In order to apply pressure more locally, a glass petri dish was placed up-side-down on the leaf (Figure 5a). Images taken at different locations in the leaf (Figure 5b and c) show that the stress response in 19 DAS old leaves is most prominent in cells located in close proximity to the applied pressure (Figure 5c, blue zone), where curved supracellular patterns could be observed. Microtubules within the cells at the extreme ends of the leaf (base and top) are not responsive (Figure 5c, green zone). In the contact zone where the petri dish touched the leaf, no clear bundling of microtubules could be observed. The stress response was also absent in 22 DAS-old leaves, even when a double load was applied.

**Table 1:** Scoring the presence of stress responses in adaxial pavement cells

	**Normal sample (3 h)**	**Detached (3 h)**	**Sample attached to plant (3 h)**	**Submerged (3 h)**	**Special chamber (6.5 h)**
**19 DAS**	✓	✕	✓	✕	✕
**20 DAS**	✓/✕	✕	✓/✕	✕	✕

Different conditions are tested for leaves of 19 and 20 DAS. The stress response, determined as the presence of strong aligned microtubules within the adaxial pavement cells as observed in the positive control (normal sample, 3 h), is scored for the 4 different conditions, whereby a ✓ marks the presence and an X the absence of stress patterns.

## Discussion

**Figure 6:**
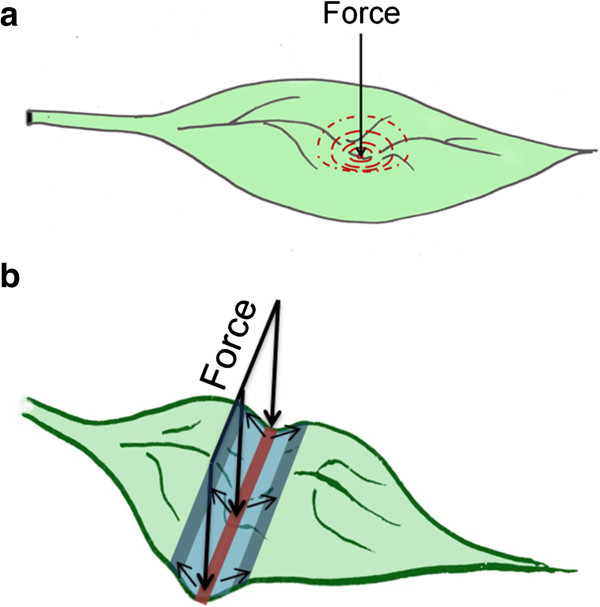
**Stress patterns in the leaf.** Image **(a)** shows the situation when a global external load is perceived by the stomata and generates a circular stress pattern. A rod-shaped application of external pressure **(b)** creates a perpendicular stress in the surrounding regions.

Microtubules form a dynamic network that is continuously subjected to polymerization, de-polymerization and pausing [20]. This constant rebuilding allows a fast and adequate response to environmental signals. A microtubule response after local mechanical stimulation with a micro needle is reported previously [21]. Here we demonstrate microtubule reorganization upon global applied pressure, which is seen as a strong alignment in parallel bundles. Moreover, microtubules between neighboring cells quite often seem to follow each other’s directions, generating a seemingly 'continuous’ supracellular pattern. When uniform pressure was exerted, by placing a cover slip on top of the leaf, a prominent feature was the resulting alignment of microtubule in circles around the stomata. A possible explanation for this pattern could be that in the TUA6-GFP line that lacks trichomes stomata which rise above the surface capture all the pressure and generate a circular stretch field for the surrounding cells (Figure 6a). Microtubules 'sense’ these changes in stresses and align according to this pattern. Similarly, when local pressure was applied in a rod-shaped form (like the petri dish) (Figure 5b), a linear front is created that may stretch the cells in a direction perpendicular to the axis of the contact area. This may also explain why no obvious changes were observed in microtubule patterns at the contact zone, since the force at this location was strictly vertical. However, there was no straightforward correlation between the stress pattern and microtubule alignment; supracellular patterns appeared curved and not following the direction of overall load application. This may implicate that the stress field is not simply linear, likely because of the cellular organization and the complex geometry of the individual cells. It is possible that stomata act as 'masts’ that cause deviated stress patterns, since stomata seem to center the supracellular patterns (Figure 5c). It would be interesting to visualize larger regions of the leaf to map the microtubule patterns and see how this may correlate with stresses in the leaf upon applied pressure.

The benefit of this particular microtubule array could lay in the subsequent modification of the orientation of cellulose microfibril deposition in the cell wall. Since the cells and moreover, the leaf tissue need to maintain their integrity, reinforcement of the wall may be needed to resist the extra endured forces. Indeed, a similar effect is discussed by Hamant *et al*. [1] for microtubules in the shoot apical meristem that respond to differential stresses, caused by cell ablation. A similar organization of microfibrils in the cells wall results in the observed anisotropic expansion perpendicular to the influential stress [22]. Upon external applied pressures, the forces in the leaf are altered thus the cells need to respond in a proper way to cope with the new conditions. This may also explain the age-dependent effect. It is reasonable that younger cells, which are actively shaping their cell walls (Jacques *et al*., unpublished results), are more vulnerable to altered mechanical stresses, while in older leaves, the walls are more robust and therefore can sustain the applied forces.

These results point to an important potential source of artifacts when studying microtuble orientation in leaves, for example for the purpose of phenotyping or developmental studies. The experimental set-up used for imaging microtubules needs to be verified carefully to ensure that the mounting of the leaves does not alter microtubule orientation within the time period of observation. A time window of maximal two hours should be respected to exclude side effects of the sample preparation when observing the Arabidopsis leaf.

## Conclusions

Nowadays, the biophysical aspects during morphogenesis are gaining more importance [23]. However, it dates back to the early seventies, when Lintilhac [24] postulated the possibility that mechanical stresses orient microtubules. Experimental data now confirm this theory ([1,2,21,25], this report). Here, we provide evidence that microtubules in pavement cells of *Arabidopsis thaliana* are responsive to applied pressures in an age-dependent manner. This encourages looking further into the potential importance of mechanical stresses in the developing leaf. Also the observation that mechanical cues may influence auxin transport [26] further stimulates research on this, until now, largely unexplored topic.

## Methods

The microtubules of the adaxial pavement cells in the fourth leaf were visualised using a fusion construct of the green fluorescent protein (GFP) tagged to α-tubulin 6 in Colombia 0 (Col-0) background (glTUA6-GFP) [27]. The leaves were cut off at the petiole from the shoot and placed between a microscope slide and coverslip in distilled water.

To test the age-effect, microtubule patterns of leaves of 15 to 20 and 22 days after sowing (DAS) were monitored during 3 hours. The cortical microtubule pattern was visualized with a Nikon C1 laser scanning confocal microscope (Nikon, Melville, NY) equipped with an argon and a helium/neon laser using a 63x planfluor lens (NA: 1.95) and the automated filter sets for GFP. A z-stack was made and the successive slides were merged into a maximal projection image, which was used for the observations.

Different conditions were tested by cutting off the leaf and placing it for 3 hours on a microscope slide or submerging it in distilled water in a glass petri dish (50 mm in diameter). Moreover, a sample was prepared with the fourth leaf still attached to the plant. Special chambers with spacers in between the microscopic slide and coverslip are prepared as described in Sawchuk *et al.*[28] to monitor leaves up to 6.5 hours (Figure 4a). A normal sample whereby the leaf was mounted in water between a microscopic slide and coverslip, acted as a positive control. Microtubules were visualized after 3 hours for all treatments with a Nikon C1 laser scanning confocal microscope (Nikon, Melville, NY). The negative control was a freshly made sample. The images were taken at the base, middle and top zone of the leaf. This was performed for leaves of 19 and 20 DAS.

A glass petri dish (60 mm in diameter) was placed on top of the leaf of 19 and 22 DAS to induce local pressure (as shown in Figure 5). The total weight of the glass petri dish was 17 g, meaning that it had a weight/circumference ratio of 0.90 g/cm. For the 22 DAS old leaves, an additional experiment was performed with an extra weight (34 g total weight). Images of the microtubules were taken in regions underneath and neighbouring the applied pressure and at more distal positions in the leaf.

Three leaves were sampled for all conditions tested and scored for the presence of stress patterns by visual observation. At least five images were taken per leaf. When in more than 50% of the images highly bundled microtubules were detected, they were positively scored for the presence of stress patterns.

## Competing interests

The authors declare that they have no competing interests.

## Authors’ contributions

EJ carried out the experiments and drafted the manuscript. EJ, JPV and KV designed the experiments and edited the manuscript. All authors read and approved the final manuscript.
